# Peripheral, Central, and Cross Sensitization in Endometriosis-Associated Pain and Comorbid Pain Syndromes

**DOI:** 10.3389/frph.2021.729642

**Published:** 2021-09-01

**Authors:** Helen C. McNamara, Helena C. Frawley, Jacqueline F. Donoghue, Emma Readman, Martin Healey, Lenore Ellett, Charlotte Reddington, Lauren J. Hicks, Keryn Harlow, Peter A. W. Rogers, Claudia Cheng

**Affiliations:** ^1^Royal Women's Hospital, Melbourne, VIC, Australia; ^2^School of Health Sciences, University of Melbourne, Parkville, VIC, Australia; ^3^Mercy Hospital for Women, Melbourne, VIC, Australia; ^4^Department of Obstetrics and Gynaecology, University of Melbourne, Parkville, VIC, Australia

**Keywords:** endometriosis, persistent pelvic pain, peripheral sensitization, central sensitization, cross sensitization

## Abstract

Endometriosis-associated pain and the mechanisms responsible for its initiation and persistence are complex and difficult to treat. Endometriosis-associated pain is experienced as dysmenorrhea, cyclical pain related to organ function including dysuria, dyschezia and dyspareunia, and persistent pelvic pain. Pain symptomatology correlates poorly with the extent of macroscopic disease. In addition to the local effects of disease, endometriosis-associated pain develops as a product of peripheral sensitization, central sensitization and cross sensitization. Endometriosis-associated pain is further contributed to by comorbid pain conditions, such as bladder pain syndrome, irritable bowel syndrome, abdomino-pelvic myalgia and vulvodynia. This article will review endometriosis-associated pain, its mechanisms, and its comorbid pain syndromes with a view to aiding the clinician in navigating the literature and terminology of pain and pain syndromes. Limitations of our current understanding of endometriosis-associated pain will be acknowledged. Where possible, commonalities in pain mechanisms between endometriosis-associated pain and comorbid pain syndromes will be highlighted.

## Introduction

Endometriosis is a complex, estrogen dependent disorder with a cumulative prevalence of 11% (1). Endometriosis is characterized by the presence of endometrium-like glands and stroma outside the uterus (2). The pathogenesis of endometriosis remains incompletely defined with genetic and environmental components contributing (3), and can be considered in terms of predisposing factors, initiating factors and propagating factors (4).

Predisposing factors are genetic and anatomical. Genetic factors have been the subject of extensive investigation. Studies have demonstrated that first degree relatives and twins of individuals with endometriosis are at increased risk of disease, and of disease of a more severe stage (5). Genome wide association studies have identified genetic variants linked with severe endometriosis (6). Genetic variants may impact inflammation, cell adhesion, growth factors and hormone receptors (7, 8). Studies using a candidate gene approach have been difficult to replicate. Anatomical factors may also contribute. Individuals with Müllerian abnormalities and/or outflow tract obstruction are at increased risk (9).

Initiating factors likely include retrograde menstruation and coelomic metaplasia. Sampson's hypothesis of retrograde menstruation proposes that endometrial fragments migrate via the fallopian tube to the peritoneal cavity (10). The increased risk of endometriosis seen with outflow tract obstruction, early menarche and short menstrual cycles may be due to an increased volume of retrograde menstruation (11). However, the mechanisms of progression to lesion formation remain unclear and only occur in a subset of women (12). Alternatively, coelomic metaplasia refers to metaplasia of cells into endometrial cells within the visceral and abdominal peritoneum. The presence of endometriosis in males exposed to estrogen, in prepubescent girls, and at extra-pelvic sites including the thoracic cavity give support to this theory (13–16). Other stem cells and progenitor cells might also initiate lesion growth either within or outside the peritoneal cavity (17).

Propagating factors are thought to include an enhanced inflammatory response, alterations in immune response including defective apoptosis, and hormonal changes including progesterone resistance (18). Endometriosis is an inflammatory condition, with increased cytokines and inflammatory mediators found in the peritoneal fluid of affected individuals (Table 1; discussed below). The development of endometriotic lesions may depend on disruption of the innate immune response to menstrual debris and ectopic endometrial cells within the pelvis, with alterations in the concentration and function of peritoneal lymphocytes and macrophages observed (19). Finally, indirect evidence exists to support a theory of progesterone resistance, predominantly related to progesterone receptor alterations within the lesions (20).

**Table 1:** Local mediators of inflammation and neuroangiogenesis in endometriosis.

**Source**	**Induce inflammation**	**Induce neuroangiogenesis**
Peritoneal fluid	Interleukin 1β Interleukin 6 Interleukin 8 Tumor necrosis factor alpha Chemokine ligand 5^*^ Chemokine ligand 2^*^ Osteoprotegerin Glycodelin	Vascular endothelial growth factor Transforming growth factor beta Nerve growth factor Brain-derived neurotrophic factor Neurotrophin-4 Semaphorins Protein gene product

* *CCL 5 = RANTES (Regulated on Activation Normal T-cell Expressed and Secreted). CCL 2 = monocyte chemotactic protein 1*.

The diagnosis of endometriosis is based on laparoscopic visualization and histology of lesions (21). Ultrasound and MRI act as adjunct methods of diagnosing deep infiltrating endometriosis and endometriomas (22). Evidence to support the use of ultrasound for the diagnosis of superficial endometriosis is emerging, but very limited (23). Clinical history and examination may aid clinicians in suspecting severe disease. However, symptom-based prediction is poor at predicting any-stage endometriosis (24). It is difficult to correlate the level of pain with the macroscopic extent of endometriosis visualized at laparoscopy (25). Some individuals with severe pain have minimal disease (26), and some individuals with severe disease have no pain (27). Laparoscopic surgical removal of endometriotic lesions reduces pain in some, but not all, individuals (28, 29). Current guidelines recommend access to a multidisciplinary pain management service as needed (30–32).

Endometriosis-associated pain (EAP) is experienced as dysmenorrhea, non-cyclical pelvic pain, and cyclical pain related to organ function including dysuria, dyschezia and deep dyspareunia (33, 34). The complexity of EAP is contributed to by the different mechanisms involved. In addition to local factors, EAP may arise and persist as a product of peripheral sensitization, central sensitization and cross sensitization (35). EAP is further contributed to by comorbid persistent pain conditions including bladder pain syndrome, irritable bowel syndrome, abdomino-pelvic myalgia and vulvodynia (36). This article will review EAP, its mechanisms, and its comorbid pain syndromes with a view to aiding the clinician in navigating the terminology of pain and pain syndromes, current knowledge, and its limitations.

## Terminology of Endometriosis-Associated Pain

Pain refers to an “unpleasant sensory and emotional experience associated with actual or potential tissue damage” (37).

Persistent pelvic pain is defined as pain perceived to be arising from the pelvis that persists >6 months' duration (38). It is acknowledged that while international societies and guidelines refer to “chronic” pelvic pain, the authors, like other groups (39–41), prefer the term “persistent” pelvic pain.

Endometriosis-associated pain has been variably described and defined either as a subset of persistent pelvic pain, or as a discrete condition. Different definitions have arisen from guidelines and classifications to facilitate and aid patient care and research, and have evolved over time. The most recent World Health Organization International Classification of Diseases (ICD-11) defines chronic primary pain syndromes, as distinguished from chronic secondary pain associated with other diagnoses (42). As a subset of chronic secondary pain, chronic secondary visceral pain may be further defined as having a causative condition like endometriosis (42).

Alternatively, the International Association for the Study of Pain (IASP) differentiates between diseases (endometriosis, secondary dysmenorrhea with endometriosis) and chronic pelvic pain syndromes, and specifically defines “endometriosis-associated pain syndrome” (37). Similarly, the European Association of Urology (EAU) separates pain syndromes with no obvious diagnosis from chronic primary pelvic pain syndromes and non-pain syndromes, and follows the IASP in separately defining “endometriosis-associated pain syndrome” (43).

As such, endometriosis-associated pain syndrome is defined as persistent or recurrent pelvic pain in individuals with laparoscopically diagnosed endometriosis, where symptoms persist after adequate treatment, and is associated with “cognitive, behavioral, sexual or emotional consequences” (43). Nevertheless, as outlined by the IASP and EAU, the phenotype of endometriosis may be less relevant. In patients with pain where endometriosis is found, it is not clear whether endometriosis is coexistent or causative (43). In addition to the fact that pain can exceed levels expected based on endometriotic lesions visualized at laparoscopy, individuals often experience symptoms of lower urinary tract, sexual and bowel dysfunction.

Therefore, while definitions are essential, they are restricted by the limitations of our current understanding of endometriosis, EAP, and its mechanisms, and by the presence of comorbid pain syndromes. For the purposes of this review, EAP refers simply to pain where endometriosis has been identified.

## Mechanisms of Endometriosis-Associated Pain

Endometriosis-associated pain arises due to initial local inflammatory and nociceptive events. It may persist as a function of peripheral, central, and cross sensitization (44). Psychosocial factors also contribute (45).

### Local Factors

The generation and sense of EAP is multifactorial, and confounded by the complexity of endometriotic lesion development, and diversity of lesion placement. Contributing factors include altered distribution of pelvic nerve fibers, inflammation, secreted factors, and neuronal growth (46–49).

Endometriosis-associated pain involves stimulation of nerve fibers of the peritoneal and visceral tissues. The parietal peritoneal tissue is highly innervated with sensory nerve fibers, sympathetic nerve fibers, myelinated and unmyelinated nerve fibers, presynaptic vesicles and neuropeptides that sense pain, pressure, touch, friction, cutting and temperature (50–52). The visceral tissue is innervated by the autonomic nervous system, with unmyelinated sensory and myelinated mechanosensory neurons that sense stretching, tearing, distention and contraction. The sensation of pain may arise from these networks. Thin unmyelinated nerve fibers lie just below the peritoneal surface (51). Myelinated nerve fibers arise from the sub-mesothelial tissue and penetrate the peritoneal cavity with demyelinated nerve endings. These nerve endings may then sense the peritoneal fluid for noxious or nociceptive stimuli (52).

Endometriotic lesions are sources of inflammation, cytokines, angiogenic factors and nerve growth factors. Inflammatory mediators at both lesion sites and within the peritoneal fluid have been investigated as potential activators of nociceptive pathways (39). Inflammatory mediators including interleukin-1β (IL-1β), interleukin-6 (IL-6), interleukin-8 (IL-8), regulated upon activation, normal T cell expressed and secreted [RANTES, or chemokine ligand 5 (CCL5)] and tumor necrosis factor alpha (TNF-α), and chemokines including monocyte chemotactic protein 1 [chemokine ligand 2 (CCL2)] are increased in the peritoneal fluid of women with endometriosis (Table 1) (53–58).

It has been commonly suggested that, consistent with endometrial tissue, endometriotic lesions cycle and bleed in response to changes in estrogen and progesterone. As such, apoptosis, necrosis and shedding would trigger inflammation and pain (35, 59–61). Cyclic changes have been thought to be responsible for hemorrhage at lesion sites visualized at the time of laparoscopy (62). However, more recent data suggest endometriotic lesions rarely cycle in synchrony with the eutopic endometrium (63). Endometriotic lesions are heterogeneous in histological morphology and hormonal responsiveness, with only some lesions demonstrating cyclic changes (64). Hemorrhage within lesions is observed throughout the menstrual cycle and may be caused by local inflammation and angiogenesis (63). Recognizing the heterogeneity of endometriotic lesions is an important step toward understanding the complexity of EAP and its treatment.

### Peripheral Sensitization

Peripheral sensitization refers to “increased responsiveness and reduced threshold of nociceptive neurons in the periphery to the stimulation of their receptive fields” (37, 59). It is proposed that persistence of EAP is associated with sensitization of the peripheral nervous system. The following mechanisms may contribute to peripheral sensitization: altered nerve density at lesion sites, changes in the peritoneal fluid, perineural invasion, and alterations in the sympathetic nervous system.

The structure and function of peripheral nerves are altered in endometriosis. Changes in nerve density have been observed in peritoneal endometriosis, ovarian endometriomas and deep infiltrating endometriosis (DIE). Significantly more nerve fibers are found in endometriotic lesions compared with normal peritoneum (65). Ovarian endometriomas have been found to have more nerve fibers compared with normal ovarian tissue (66). DIE has a higher nerve fiber density compared with endometriosis at other sites; endometriosis in the rectovaginal septum has been demonstrated to have higher nerve fiber density compared with superficial peritoneal endometriosis (55, 67). Moreover, increased density of calcitonin gene-related peptide (CGRP) positive nerve fibers in lesions has been shown to positively correlate with pain severity in individuals with endometriosis (68).

Differences in receptors on peripheral nerve fibers have also been observed. The density of transient receptor potential vanilloid 1 receptor (TRPV1), involved in nociceptive pain pathways, is increased in endometriotic lesions, and its increased density has been associated with increasingly severe dysmenorrhea (69).

Alterations in the peritoneal fluid may cause sensitization of peripheral nociceptors. Changes in peritoneal fluid contribute to neuroangiogenesis in endometriotic lesions and in the adjacent peritoneum. Neurotrophins [nerve growth factor (NGF), brain derived neurotrophic factor (BDNF), neurotrophin-4] and other growth factors [vascular endothelial growth factor (VEGF) and transforming growth factor beta (TGF-β)] are increased in the peritoneal fluid of women with endometriosis (Table 1). Growth factors stimulate new nerve growth in the periphery, potentially contributing to the altered nerve density in endometriotic lesions (70).

Perineural invasion (PNI) may also be associated with pelvic sensitization. PNI, more commonly associated with malignancy, is defined in this context as the migration or integration of endometriotic lesions along nerve fibers (71–73). PNI in patients with deep infiltrative endometriosis has been shown to be associated with higher pain scores, dysmenorrhea, dyspareunia, persistent pelvic pain, sciatica and unilateral leg pain (73, 74). PNI contributes to increased neurogenesis and angiogenesis (75). Increased endometriotic lesion secretion of NGF (76) and TGF-β (77) also correlates with lesional invasion of pelvic nerves (70).

Dysregulation of the autonomic nervous system (ANS) may further amplify peripheral sensitization. Some endometriotic lesions are found to have a lower density of sympathetic nerve fibers while sensory nerve fiber density is unchanged (78). A loss of sympathetic nerve fibers is associated with chronic inflammation, and is seen in other chronic inflammatory diseases (79). However, the mechanisms by which ANS dysregulation contributes to EAP remain unclear.

### Central Sensitization

Central sensitization refers to the heightened excitability of the central nervous system in response to noxious stimuli (37). Central sensitization has been classically described as increased excitability of nociceptive neurons in the dorsal horn of the spinal cord following continued or recurrent exposure to noxious stimuli, tissue injury or nerve damage (80). Changes in dorsal horn neurons are exhibited as a decreased pain threshold and an increased pain response. Subsequent alterations in synaptic efficacy in surrounding nerves gives rise to central facilitation, with pain experienced due to innoxious stimuli. Impaired descending pain modulation at the level of the dorsal horn neurons may also contribute to central sensitization (81).

It has been demonstrated that women with endometriosis and persistent pelvic pain demonstrate hyperalgesia in response to noxious stimuli compared to healthy pain-free controls (82). Evidence is mixed when women with pelvic pain with endometriosis and women with pelvic pain without endometriosis are compared, with studies finding increased hyperalgesia in those with biopsy proven endometriosis (82), or no difference (40).

Central sensitization may also be described as arising due to primary changes in brain activity or structure. Changes in brain structure, function and activity have been evaluated in individuals with persistent pain using functional MRI and PET imaging (83). Women with EAP have been found to have reduced volume in areas of the brain associated with pain processing, including the thalamus, insula and putamen (84). It is suggested that reduced tissue volume occurs due to neuronal atrophy, neurodegeneration or medication effects (44).

Using functional MRI, it has been demonstrated that women with EAP have increased resting-state connectivity between pain processing regions including the anterior insula compared to women with endometriosis and no pain, and healthy controls (85). Women with dysmenorrhea have been found to have increased activation of pain processing regions in response to noxious stimuli both within the pelvis and in the periphery (86).

Finally, central sensitization may occur as a consequence of changes in the hypothalamic-pituitary-adrenal (HPA) axis. Women with persistent pelvic pain display alterations in their HPA axis and HPA axis-mediated pain response. Persistent pain alters the HPA axis and its capacity to mount a stress response to noxious stimuli. Women with dysmenorrhea have been demonstrated to have reduced levels of cortisol compared to women without pain (86). Acute stress leads to activation of the HPA axis but with chronic insult, this response hypo-attenuates (44).

### Cross Sensitization

Cross sensitization refers to nociceptive inputs from a diseased tissue impacting the perception of pain arising from normal tissue in close proximity (87). Cross sensitization has been studied between the pelvic organs of the bladder, colon, uterus and vagina. It is suggested that sensitized afferent nerves of the uterus and vagina give rise to sensitization in visceral afferents of other organs. These peripheral afferent nerve pathways overlap, converging on similar areas of the spinal cord. This is referred to as viscero-visceral convergence. Cross sensitization has also been observed between pelvic floor muscles and pelvic viscera. This is referred to as somato-visceral convergence (88).

It is hypothesized that shared innervation pathways have evolved to facilitate the coordination of pelvic organ functions—urination, defecation and sexual function—and that the presence of endometriosis causes maladaptation of converging pain pathways. In endometriosis, neurogenesis within lesions may also play a role, with newly sprouted nerve fibers converging on existing innervation pathways (35).

Viscero-visceral hyperalgesia refers to increased pain experienced by women with endometriosis *and* an associated pelvic pain syndrome. Viscero-visceral hyperalgesia is thought to contribute to pain severity in women with endometriosis (89).

### Psychosocial Factors

Pain processing is further influenced by genetics, psychological state and cognitive factors (44). Women with persistent pelvic pain and endometriosis report high levels of mood disorders, with population-based studies demonstrating a bidirectional association of diagnosis with endometriosis and psychiatric disorders (90). The mechanisms underlying these associations are complex (91), and impacted by individuals' perception of social functioning (92). Subsequent alterations in pain perception have been observed (93–95). Depressed mood, anxiety, pain catastrophizing, pain anticipation and increased attention to pain are associated with higher pain intensity (39). Evidence suggests that psychosocial factors impact pain processing at all levels, local, peripheral and central (44).

## Comorbid Pain Syndromes

Endometriosis-associated pain often coexists with, and is further complicated by, other pelvic pain syndromes including bladder pain syndrome, irritable bowel syndrome, vulvodynia and abdomino-pelvic myalgia (92). Comorbid pain syndromes have mechanistic features in common with EAP, including peripheral, central, and cross sensitization.

It is acknowledged that while common comorbid *pelvic* pain syndromes are reviewed in this article, other “chronic overlapping pain conditions” including migraine, lower back pain and fibromyalgia are beyond the scope of this review (Table 2) (96).

**Table 2:** Comorbid pain syndromes vs. chronic overlapping pain conditions.

**Comorbid pain syndromes**	**Chronic overlapping pain conditions**
Endometriosis-associated pain	Endometriosis
Persistent pelvic pain	Chronic tension headache
Painful bladder syndrome	Migraine
Irritable bowel syndrome	Temporo-mandibular joint disorder
Provoked vulvar vestibulodynia	Chronic lower back pain
Abdomino-pelvic myalgia	Fibromyalgia
Viscero-visceral hyperalgesia syndrome	Chronic fatigue syndrome.

*Table compiled using data from Chronic Pain Research Alliance (96) and Affaitati et al. (89)*.

### Bladder Pain Syndrome

Bladder pain syndrome (BPS) is characterized by pain in the bladder and/or pelvis associated with lower urinary tract symptoms including urinary urgency and frequency (97). In women diagnosed with endometriosis at laparoscopy the rate of co-existing BPS is in the order of 43–60% (98–100). Women with endometriosis have a 4-fold risk of diagnosis with BPS within 3 years of diagnosis with endometriosis (101). Like EAP, pain in BPS is mediated by inflammation. High levels of urothelial inflammatory mediators including IL-6 and TNF-α are observed in individuals with BPS (102). Central pain amplification and altered processing of afferent signals may also contribute (103).

### Irritable Bowel Syndrome

Irritable bowel syndrome (IBS) is a functional gastrointestinal disorder defined by the Rome IV criteria as the presence of abdominal pain related to defecation and/or change in bowel habit, in the absence of other gastrointestinal disease (104). In women diagnosed with endometriosis the rate of IBS is as high as 60% (105). In a cohort of adolescents with endometriosis, 24% self-reported a concurrent diagnosis of IBS, with an increased association between pain severity and diagnosis with IBS (106). The pathogenesis of IBS is thought to relate to alterations in the enteric nervous system and brain-gut interactions. Peripheral sensitization may contribute with similar mechanisms of sensitization observed in IBS as to those seen in EAP, including increased activation of the TRPV1 receptor (107). Altered visceral sensation and pain in IBS may also be a consequence of central sensitization, with changes observed in the activation of brain regions associated with emotional arousal and pain modulation in individuals with IBS (108).

### Abdomino-Pelvic Myalgia

Abdomino-pelvic myalgia refers to pain felt in the abdominal or pelvic muscles and surrounding connective tissue. Myalgia refers to tenderness (allodynia or hyperalgesia) of the muscles on palpation, in the absence of increased tone (109). If increased muscle tone is detected, the condition is termed abdominal or pelvic tension myalgia, and further defined as pelvic floor muscle (PFM) tension myalgia if the location of the tenderness and tone are specific to the PFM (109). Women with EAP are highly likely to be found to have PFM tension (110). PFM tension is more common in those with biopsy proven endometriosis than in women with persistent pelvic pain without endometriosis (82). Individuals with DIE have been found to have PFM tension in 29%, with inappropriate and weak PFM contraction, and inability to completely relax in up to 45% (111). Both peripheral and central nervous system changes have been implicated in the development of myalgia (112). Peripheral nociceptors in pelvic floor muscles may contribute to referred pain through central mechanisms, at the level of the dorsal horn (113).

### Vulvodynia

Vulvodynia refers to vulvar pain of at least 3 months' duration, without clear identifiable cause (114). The most commonly reported subtype is provoked vestibulodynia (PVD) where pain is provoked and localized to the vulvar vestibule. Diagnostic criteria sometimes include positive cotton swab testing. PVD gives rise to superficial dyspareunia and has a negative impact on sexual functioning (114). Vulvodynia and endometriosis are comorbid in ~11% of affected individuals (115, 116). PVD has been diagnosed in individuals with persistent pelvic pain (with or without a diagnosis of endometriosis) in 33% (117). Moreover, PVD is reported in up to 74% of individuals with BPS (118). Central sensitization may contribute. Individuals with PVD exhibit decreased pain thresholds in regions distant to the vulva including the thumb and deltoid (119, 120). Studies related to changes in brain structure in individuals with PVD demonstrate mixed results, with a trend toward increased gray matter in pain modulating regions in younger women and decreased gray matter in older women (121). In pre-menopausal women, increased gray matter volume has been observed in the basal ganglia, hippocampus and sensorimotor cortices (122). PVD is associated with psychological distress including pain related anxiety and depression (123).

## Conclusion

Endometriosis-associated pain and the mechanisms responsible for its initiation and persistence are complex. Therefore, EAP is difficult to treat. The frequent presence of comorbid pelvic pain syndromes adds to the difficulty. In this review, the terminology and mechanisms of EAP have been described. Mechanisms that are common between EAP and comorbid pain syndromes have been highlighted. It is clear that peripheral sensitization, central sensitization and cross-sensitization are important future therapeutic targets. It is prudent that clinicians attend to the biological, psychological and social factors contributing to individuals' experience of EAP, and screen for comorbid pelvic pain syndromes.

## Author Contributions

HF and CC conceived and designed the study. HM wrote the original manuscript. JD, ER, MH, LE, CR, LH, KH, and PR made comments and contributed to subsequent drafts of the manuscript. All authors contributed to the article and approved the submitted version.

## Conflict of Interest

The authors declare that the research was conducted in the absence of any commercial or financial relationships that could be construed as a potential conflict of interest.

## Publisher's Note

All claims expressed in this article are solely those of the authors and do not necessarily represent those of their affiliated organizations, or those of the publisher, the editors and the reviewers. Any product that may be evaluated in this article, or claim that may be made by its manufacturer, is not guaranteed or endorsed by the publisher.
